# CFRP Reinforced Foam Concrete Subjected to Dynamic Compression at Medium Strain Rate

**DOI:** 10.3390/ma13010010

**Published:** 2019-12-18

**Authors:** Xiaojuan Wang, Lu Liu, Wenjing Shen, Hongyuan Zhou

**Affiliations:** Key Laboratory of Urban Security and Disaster Engineering of Ministry of Education, Beijing University of Technology, Beijing 100124, China; xiaojuanwang@bjut.edu.cn (X.W.); liul@emails.bjut.edu.cn (L.L.); e-newton@163.com (W.S.)

**Keywords:** CFRP confinement, foam concrete, energy absorption, protective structure

## Abstract

Carbon fiber-reinforced polymer (CFRP)-confined foam concrete can be applied in structure protection, e.g., as an impact barrier of bridge piers, in which it is used as the core of the composite impact barrier. Applying CFRP to the foam concrete exterior enhances both the CFRP and the foam concrete, leading to improved compressive performance due to their interaction. In the present study, the carbon-fiber reinforced polymer (CFRP) confining effect on the response and energy absorption of foam concrete subjected to quasi-static and medium-strain-rate dynamic compression was experimentally investigated. The confinement by CFRP changed the response and failure mode of foam concrete specimens from shear in quasi-static load and splitting in dynamic load to crushing, resulting in a significant increase in the load bearing and energy absorption capacity. The composite consisting of CFRP and foam concrete was sensitive to strain rate. In particular, the CFRP–foam concrete interaction led to the remarkably improved resistance and energy absorption capacity of CFRP-confined specimens, which were significantly higher than the sum of those of standalone CFRP and foam concrete.

## 1. Introduction

The application of carbon fiber-reinforced polymer (CFRP) on foam concrete exterior remarkably improves its crushing resistance due to the confinement effect, leading to significantly increased energy absorption capacity. In engineering practice, CFRP-confined foam concrete can be applied in structure protection, e.g., as an impact barrier of bridge piers, in which it is used as the core of the composite impact barrier. The crushing absorbs a considerable amount of energy, and, at the same time, it reduces the load transferred to both the bridge and the vehicles. Moreover, the energy absorption capacity and crushing resistance can be altered by changing the foam concrete density.

Cellular solids are a group of materials with a large portion of air pores inside the base materials, usually categorized as open-cell and close-cell types due to macrostructure. While some cellular solids are ductile (e.g., metal foams, polymeric foams), some cellular solids are brittle (foam concrete and foam ceramic). In addition to their superior properties such as being lightweight and low thermal conductivity, they experience a relatively long stress plateau of nearly constant stress level with increasing strain when subjected to compression, implying excellent capacity in energy absorption [1,2,3,4]. This makes such materials efficient energy absorbers, promising in potential applications in structure protection with dynamic load [5,6,7,8,9]. 

Foam concrete is traditionally used as a thermal insulation material [10] and non-load- bearing parts of structures and buildings, such as lightweight roofs and floors [11,12,13,14]. As a typical cellular material, although few exist to date, there are increasing mechanical load-related applications of foam concrete. For instance, the overrun of planes in airport runways can be effectively stopped through the dynamic crushing of foam concrete as a special part of the runway [15]. In addition, it was found that reasonably designed foam concrete effectively mitigated tunnel lining deformation when subjected to an internal explosion [16].

The preparation and quasi-static performance of foam concrete was extensively studied [17,18], which allowed the influence of base material, relative density, and reinforcement on strength to be discussed [10,11]. Bagheri and Samea [19] further investigated the effect of air content on the rheology of foam concrete. Unlike normal-strength foam concrete, the strength of fiber-reinforced foam concrete made from high-strength concrete of density 1000–1500 kg/m^3^ and polypropylene fibers can reach up to 50 MPa [20], even higher than that of normal concrete, facilitating its application as load-bearing elements. In addition to the conventional compression test and finite element modeling at the macroscopic scale, the discrete method was employed to simulate the microscopic response and failure of foam concrete under quasi-static compression [21]. At the structural member scale, foam concrete slabs and panels were produced and tested [22].

The increasing applications of foam concrete in load bearing-related applications require a thorough understanding of its response under compression load, both static and dynamic. While the performance of foam concrete subjected to quasi-static load was extensively investigated [10], its behavior under dynamic load remains to be further understood, due to the limited literature available. The split Hopkinson pressure bar (SHPB) was employed to investigate the dynamic response of foam concrete with a relatively high strain rate [23]. Furthermore, the dynamic compression behavior of foam concrete and stress wave propagation was examined with a shock tube [24], in which the foam concrete sample was loaded with a blast generated by a sudden release of pressurized gas. With a gas gun, the penetration resistance of foam concrete subjected to an impacting projectile of mass around 20 g and velocity 10–150 m/s was examined [25]. It was verified that the rigid-perfect plastic-locking model can be used as a penetration resistance function.

The dynamic characteristics of materials including foam concrete can be investigated with testing facilities of varying loading rate. In addition to the servo-hydraulic universal machine for quasi-static and very-low-speed loading, SHPB is capable of compressively loading specimens with a relatively high strain rate, up to 10^3^ /s. While the specimens can be loaded with almost constant strain rate if properly designed, due to the mechanism of SHPB, it is difficult to obtain the complete stress–strain relationship incorporating the long stress plateau [26]. In the intermediate strain rate range, complete stress–strain curves can be obtained with drop-weight test, but the loading strain rate is not rigorously constant. To this end, a high-velocity testing machine with constant loading speed is employed to characterize the dynamic properties of foam concrete.

In the present study, the response, failure mode, and energy absorption of foam concrete with and without CFRP confinement subjected to quasi-static and dynamic loading were experimentally investigated and compared. Under different loading rates, the crushing strengths of foam concrete and CFRP wrap were tested separately. Then, the CFRP-confined foam concrete was tested quasi-statically and dynamically with corresponding loading rates to investigate the strengthening effect due to the CFRP–foam concrete interaction in terms of crushing strength. Subsequently, the energy absorption of foam concrete with and without CFRP reinforcement subjected to various loading rates was examined, and the factors determining the strengthening effect were identified and discussed.

## 2. Specimens and Test Programs

### 2.1. Specimens

**Figure 1:**
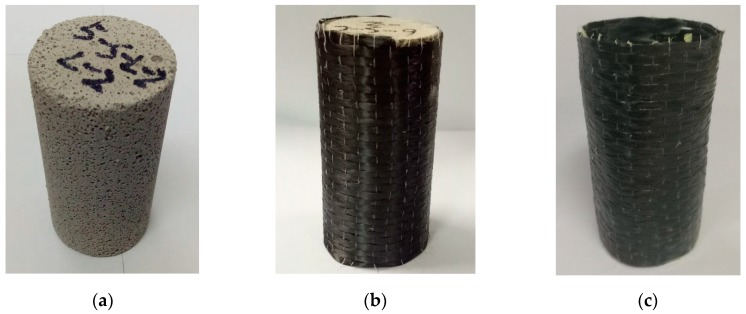
Foam concrete specimens (**a**) without and (**b**) with carbon fiber-reinforced polymer (CFRP) reinforcement; (**c**) standalone CFRP.

In the present study, the foam concrete was made by mixing C30 cement with a plant protein foaming agent (a typical physical foaming agent), without any other ingredients. Foam concrete was produced under controlled conditions from cement, water, and a liquid chemical that was diluted with water in a volume ratio of 1:40 and aerated to form the foaming agent. The cement used in the tests was common Portland cement of compressive strength 30 MPa. A pre-foaming method was used to produce foam concrete comprising the produced base mix and stable pre-formed foam, which were blended thoroughly. The 100-mm cube standard blocks were cured for 28 days. All specimens were of the same geometry and size: a cylinder of diameter 40 mm and height 80 mm, cut from 100-mm cubes, as shown in Figure 1. The cylindrical specimens were cut from the 100-mm standard cubes. Specifically, although the foam concrete is non-conductive, the specimens were cut with a special “wire-cutting” method by a cutting professional. The accuracy of cutting was 0.1 mm, and the diameter of specimens was 40 ± 0.1 mm. Both upper and lower surfaces of specimens were ground to ensure they were parallel. The densities of the specimens were roughly 400 kg/m^3^, 500 kg/m^3^, and 600 kg/m^3^. The size of the specimens was determined based on two factors. On one hand, the specimens should not be big, to make sure the loading capacity of the high-speed testing machine is not exceeded, especially under relatively high loading speeds such as 10 m/s. On the other hand, the specimens should have an adequate height-to-diameter ratio, to eliminate the friction end effect from the crosshead and the supporting anvil, according to St. Venant’s principle. In fact, it is difficult to accurately control the density of the foam concrete during the manufacturing process. Therefore, in the analysis, the density of each specimen is provided to facilitate comparisons of crushing strength and energy absorption.

The CFRP of uni-axial tensile strength 3800 MPa and elastic modulus 230 GPa was manufactured by Nanjing Haituo Company, one of the major CFRP-related product providers in China. The CFRP-reinforced foam concrete specimens were made by wrapping and gluing the CFRP around the cylindrical foam concrete specimens, whose overlapping area was half the cylinder circumference.

### 2.2. Test Programs

**Figure 2:**
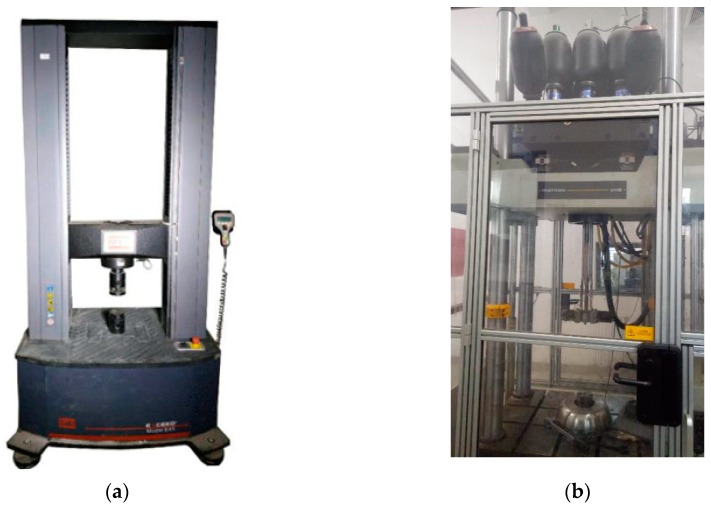
Testing machines for: (**a**) quasi-static compression; (**b**) dynamic compression.

The foam concrete with and without CFRP reinforcement, as well as the CFRP wrap alone, was subjected to both quasi-static - and dynamic compression. MTS Exceed E45 (MTS Systems Corporation, Shenzhen, China) with a maximum loading capacity of 300 kN in the structure lab of Beijing University of Technology, shown in Figure 2a, was employed to conduct the testing, at a loading rate of 5 mm/min. With a similar approach, a dynamic compression test was carried out in the structure lab of Tianjin University, where the INSTRON VHS 160/100-20 (Instron Corporation, Norwood, MA, United States) was used, shown in Figure 2b. The machine can load specimens with a constant rate at a maximum of 10 m/s for compression and 25 m/s for tension, provided that the loading force does not exceed 100 kN (maximum loading capacity). For each loading speed (1 m/s, 5 m/s, and 10 m/s), several specimens were tested, including different densities and with/without CFRP.

## 3. Results and Discussions: Quasi-Static Response and Energy Absorption

The plateau stress and densification strain are two important parameters for cellular solids such as foam concrete, determined as shown below. The energy absorption efficiency is widely accepted to be defined as

(1) $$E=\frac{1}{\sigma (\varepsilon )}\int_{0}^{\varepsilon }\sigma (\varepsilon )d\varepsilon .$$

The maximum of the efficiency with respect to strain is determined as

(2) $$\frac{d}{d\varepsilon }{\left[\frac{1}{\sigma (\varepsilon )}\int_{0}^{\varepsilon }\sigma (\varepsilon )d\varepsilon \right]|}_{\varepsilon =\varepsilon_{d}}=0.$$

The strain corresponding to the maximum energy absorption efficiency is defined as densification strain. The specific energy absorption is calculated as the area below the stress–strain relationship from zero to densification strain. In the present study, the discrete stress–strain data in pairs were used to calculate the specific energy absorption. Specifically, summing up all the products of stress readings and the corresponding small strain increment yielded the specific energy absorption. Subsequently, the plateau stress was calculated as the ratio of the specific energy absorption to densification strain.

The test results of foam concrete with and without CFRP reinforcement subjected to quasi-static compression are listed in Table 1.

**Figure 3:**
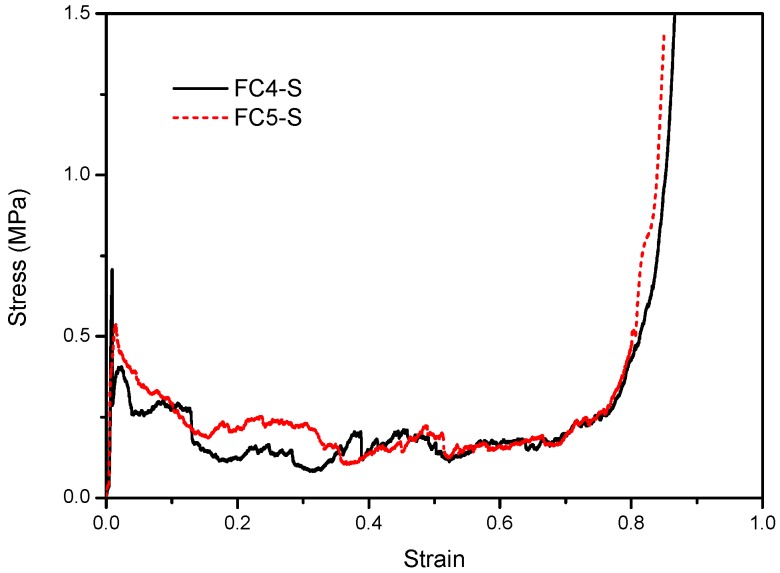
Stress–stain relationships of foam concrete subjected to quasi-static compression.

The foam concrete cylinders were compressed, and the quasi-static stress–strain relationships are shown in Figure 3. It is noteworthy that, unlike the curves of metal foam without initial overshoot peak stress, foam concrete exhibited an obvious peak stress prior to the long stress plateau, implying sudden brittle failure. The observations agree well with similar compression tests of foam concrete with different base materials [27], in which metal tailing concrete was used to manufacture the foam concrete and a relatively small strain of around 8% was crushed. Upon being subjected to further increased crushing beyond the densification strain, the walls of the foam concrete cells gradually came into contact with each other, resulting in a sharply increased compression resistance (stress).

**Figure 4:**
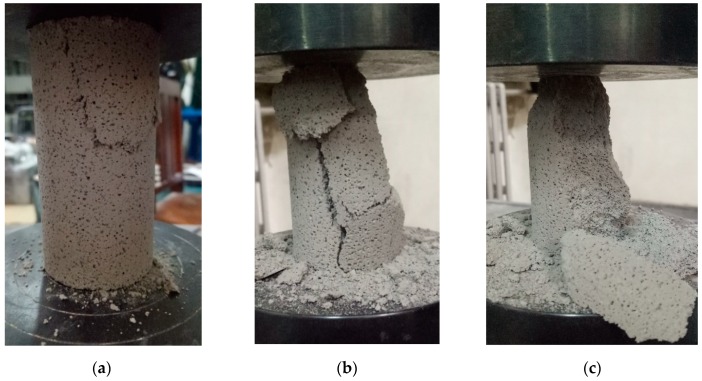
Test result of foam concrete subjected to quasi-static compression: (**a**) initial shear crack (ε = 0.008); (**b**) through-thickness crack (ε = 0.20); (**c**) detachment of large parts (ε = 0.25).

The typical response and failure mode of foam concrete is shown in Figure 4. While some parts near the loading platen were sheared off initially, major vertical cracks formed and developed with the increasing loading, followed by large parts detaching in the advanced stage.

**Figure 5:**
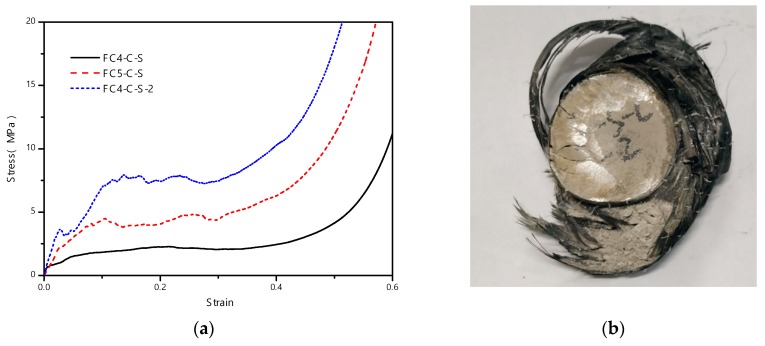
The CFRP-confined foam concrete subjected to quasi-static compression: (**a**) stress-strain relationship; (**b**) failure mode.

Figure 5 shows that, with CFRP confinement, the plateau stress significantly increased to several times that of the corresponding foam concrete without CFRP reinforcement. There was no obvious initial stress peak, which was unlike the foam concrete without CFRP. Without CFRP confinement, the initiation and development of lateral cracks during the loading process led to peak stress drops prior to the long plateau. On the contrary, the lateral cracks of foam concrete were suppressed due to CFRP confinement, resulting in no stress drop. It is noteworthy that the CFRP-confined foam concrete specimen eventually failed in the lower part of the CFRP, with fracture in the side of a single layer. The final state of the specimen is shown in Figure 5b.

As the confinement or constraint induced by the CFRP enhances the plateau stress, it is interesting to check the further enhancement if the single-layer CFRP is changed to a double layer. According to the test result shown in Figure 5a, increasing CFRP confinement led to higher plateau stress and smaller densification strain. 

**Figure 6:**
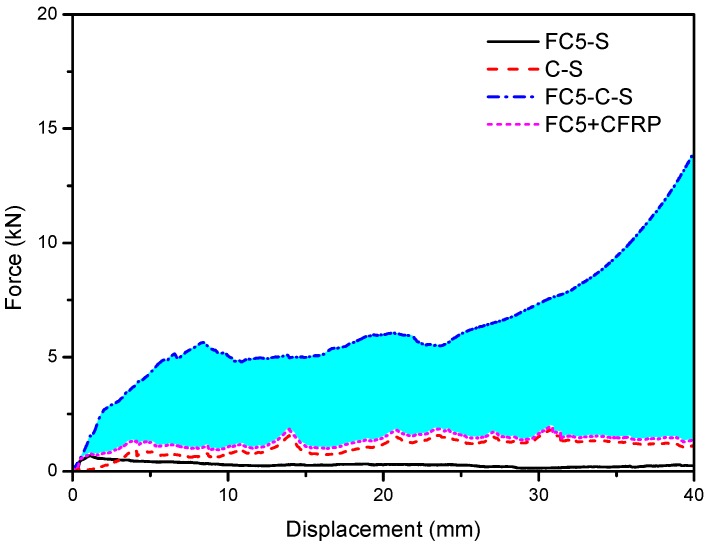
The strengthening effect due to CFRP-foam concrete interaction subjected to quasi-static compression. The blue area is the extra energy absorption due to the interaction.

The standalone CFRP wrap, without any filling, was compressed quasi-statically, whose load-bearing capacity is indicated by the red dashed line in Figure 6. More importantly, this figure shows the reinforcement effect of the CFRP on foam concrete in terms of the plateau stress and energy absorption. It is evident that the crushing resistance of CFRP-reinforced foam concrete was several times higher than the total load taken from the foam concrete and CFRP wrap compressed separately. Subsequently, the energy absorption capacity significantly improved with the same crushing deformation. The blue area in the figure denotes the extra energy absorption due to the CFRP confinement. However, the densification strain of the specimen with CFRP was reduced compared to that without CFRP. 

There were two major mechanisms for the remarkably improved plateau stress. On one hand, due to the confinement from the CFRP, the splitting along the loading direction and shear cracking was suppressed, and the failure was dominated by the crushing mode, resulting in higher resistance. On the other hand, with respect to the energy absorption, the interaction between the foam concrete and fiber wrap expanded the CFRP circumferentially, in which a considerable amount of strain energy was stored.

## 4. Results and Discussions: Dynamic Response and Energy Absorption

When it comes to the foam concrete with and without CFRP subjected to dynamic compression, the response becomes more complicated due to varying loading rate. Based on the fact that the stress of foam concrete increases sharply with increasing strain when the densification strain is reached, while taking into account that the strain rate effect induced higher resistance, the nominal strain of dynamic compression on foam concrete specimen with and without CFRP reinforcement was determined as 0.5, to avoid overloading the testing machine.

Unlike the universal testing machine with quasi-static loading and split Hopkinson pressure bar with relatively high loading rate (generally with a strain rate from several hundred to thousand s^−1^), the INSTRON VHS 160/100-20 can load specimens with intermediate strain rate in between. One of the advanced features of this machine is that, with a specially designed fixture, the specimens can be compressed with (almost) constant speed from the initial loading. In the present study, the loading speeds were chosen as 1 m/s, 5 m/s, and 10 m/s, for all foam concrete specimens with and without CFRP reinforcement. The test results are in Table 2.

**Figure 7:**
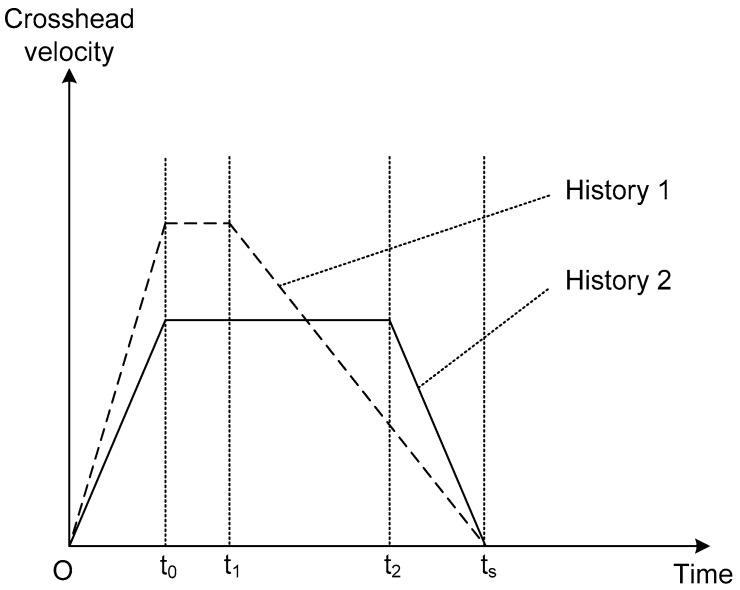
Illustration of the crosshead time histories of two designed loading speeds.

Limitations do exist in the present experimental study. As stated previously, since the loading capacity of the dynamic testing machine 100 kN cannot be exceeded and a certain safety margin must be applied in the test, the compression distance of all specimens was determined as 40 mm, half the original specimen height. Further insight into the loading velocity control of the testing machine indicated the limitation of the “constant rate loading” in practice, as shown in Figure 7. Through a special design, the crosshead cannot load the specimen before the designed loading speed is achieved, corresponding to the period from the time zero to t_0_. Then, the crosshead starts to compress the specimen for a duration with (almost) constant loading rate, i.e., from t_0_ to t_1_ for History 1 or from t_0_ to t_2_ for History 2. After that, the crosshead decelerates and finally comes to a stop at t_s_, during which the loading speed is not constant. The compression stroke for all specimens is designed the same regardless of the constant loading velocity, i.e., the area from t_0_ to t_s_ remains fixed in Figure 13, which implies that the time of constant loading for higher velocity should be shorter than that for lower velocity. This mechanism accounts for the observation that, in the initial loading stage, the plateau stress under 10 m/s was significantly higher than that under 5 m/s, while, in the advanced loading stage, the plateau stress of 10 m/s was similar to or even lower than that under 5 m/s. In fact, the initial loading stage corresponded to the constant rate loading, while the later stage corresponded to the decelerated rate loading.

**Figure 8:**
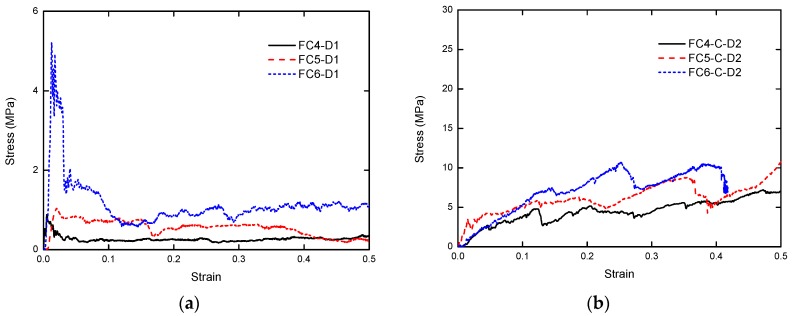
The stress–strain relationship subjected to 1-m/s compression: (**a**) foam concrete without CFRP, and (**b**) foam concrete with CFRP.

The stress–strain relationship subjected to 1-m/s compression is shown in Figure 8. Recalling Figure 3 and Figure 5, under the same strain rate, a higher density of the foam concrete led to a higher stress plateau. Similar to quasi-static loading, the dynamic plateau stress of foam concrete with CFRP was several times that of the corresponding dynamic plateau stress without CFRP. 

**Figure 9:**
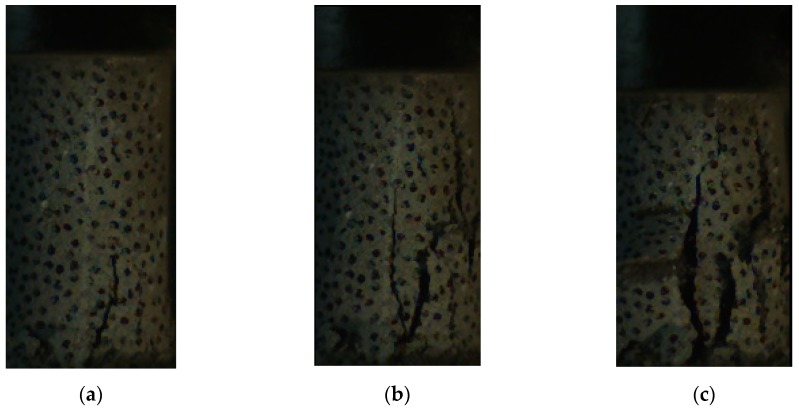
Response and failure mode of 600-kg/m^3^ foam concrete specimen subjected to 5-m/s compression. (**a**) ε = 0.05, (**b**) ε = 0.075, and (**c**) ε = 0.10.

**Figure 10:**
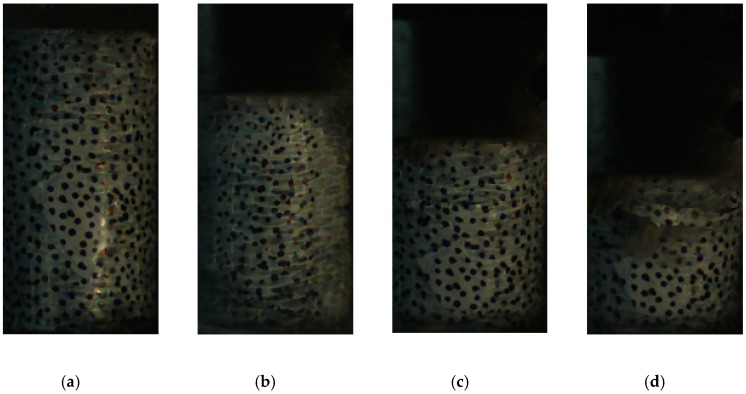
Response and failure mode of 400-kg/m^3^ foam concrete with CFRP subjected to 5-m/s compression. (**a**) ε = 0.10, (**b**) ε = 0.25, (**c**) ε = 0.35, and (**d**) ε = 0.45.

**Figure 11:**
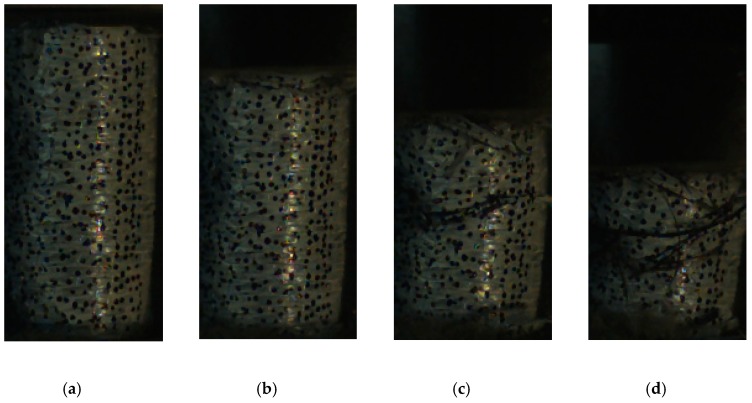
Response and failure mode of 600-kg/m^3^ foam concrete with CFRP subjected to 5-m/s compression. (**a**) ε = 0.15, (**b**) ε = 0.25, (**c**) ε = 0.35, and (**d**) ε = 0.45.

With respect to the response and failure mode, the 600 kg/m^3^ foam concrete with and without CFRP reinforcement subjected to 5 m/s crushing was taken as an example. Figure 9 shows the specimen response; without CFRP reinforcement, small axial cracks initiated at the supporting anvil, then developed and propagated upward, eventually leading to major fractures and specimen failure. With CFRP, the specimen remained intact during compression until the CFRP ruptured. In some cases, there was no fold formed during the loading, and the CFRP ruptured in the middle, as shown in Figure 10, while, in some other cases, folds formed and the CFRP ruptured in the bottom, as shown in Figure 11.

**Figure 12:**
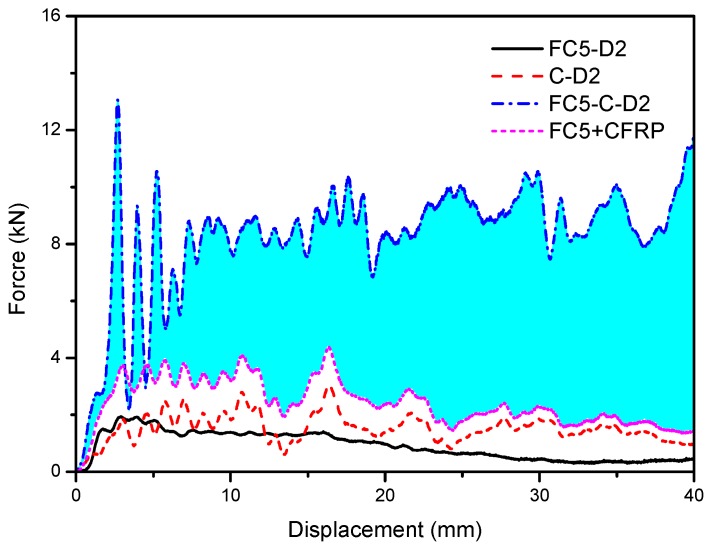
The strengthening effect of the CFRP–foam concrete interaction subjected to dynamic compression. The blue area is the extra energy absorption due to the interaction.

Subjected to dynamic load, the interaction between the foam concrete specimen and CFRP is indicated in Figure 12. Compared to Figure 6 for quasi-static loading, the strengthening effect was similar in the sense that the crushing resistance of CFRP-reinforced foam concrete, combining the foam concrete and CFRP with stronger interaction, was significantly higher than that of the standalone foam concrete and CFRP wrap simply added together. The difference from that subjected to quasi-static compression was that all stresses become higher, including the resistance of crushing foam concrete and CFRP wrap alone and the CFRP reinforced specimen. It can be observed that there are oscillations in almost all of the load–displacement curves in the figure, which is due to the lateral vibration of the crosshead during vertical compression induced by the eccentricity of the CFRP-confined specimen.

**Figure 13:**
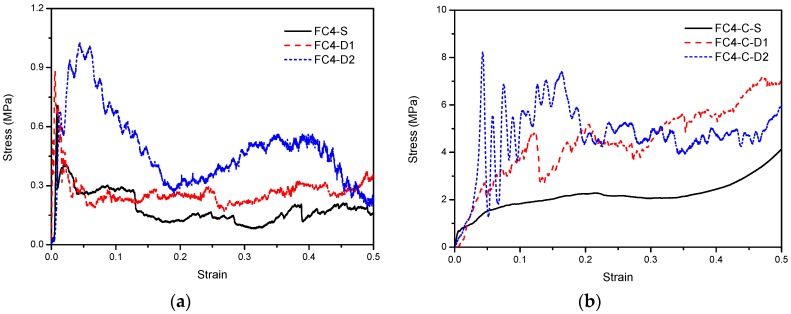
Stress–strain relationship of foam concrete with and without CFRP subjected to compression of different loading rates: (**a**) without CFRP; (**b**) with CFRP.

With increasing loading rate, the response and plateau stress of foam concrete with and without CFRP reinforcement was compared, as shown in Figure 13. In the current study, the loading rate was represented by the nominal strain rate, which is defined as the loading velocity divided by the original specimen height. In fact, the specimen response may initiate and propagate from a local part, rather than uniform global deformation. With the height of the specimens throughout the test being the same as 80 mm, the nominal strain rate was used; loading velocities were from 1 m/s to 10 m/s, and the strain rate ranged from 12.5 /s to 125 /s, representing an intermediate strain rate. It can be observed from both situations that a higher nominal strain rate led to a higher plateau stress, regardless of whether CFRP was applied to the foam concrete or not.

In the analysis of the foam concrete behavior subjected to quasi-static and dynamic loading, the energy absorption with and without CFRP reinforcement was calculated with a pre-defined nominal strain of 0.5, rather than the actual densification strain corresponding to the maximum energy absorption efficiency. Therefore, for those with a densification strain greater than 0.5, the energy absorption had the same characteristics with the plateau stress, as the concerned specific energy absorption (defined as the energy absorption divided by the undeformed volume of specimen) was simply the integral of the stress over the strain from zero to 0.5. For those with densification strain smaller than 0.5, generally foam concrete of relatively high density or with CFRP reinforcement, the actual densification strain should be used for calculating the specific energy absorption. In fact, in addition to absorbing a considerable amount of energy, the foam concrete should control the transferred load to a designed level regardless of strain in the application range, to facilitate protective structure design in engineering practice.

While foam concrete is relatively cheap, CFRP is relatively expensive in structural engineering applications. However, applying CFRP significantly improves the protection performance. To achieve the same load bearing and energy absorption capacity, the amount of foam concrete usage can be remarkably reduced if CFRP is applied to the exterior of the foam concrete, which has multiple merits. On one hand, the core size of foam concrete protective structures is reduced, facilitating the design of such structures. On the other hand, it is more environmentally friendly as the usage of concrete is reduced.

## 5. Conclusions

Foam concrete with and without CFRP reinforcement subjected to quasi-static and dynamic compression was experimentally investigated, in which the resistance and energy absorption capacity were examined. From the test results, the following findings can be reached:

1. The response and failure modes of foam concrete varied from shear and splitting without CFRP to crushing by applying CFRP confinement;

2. The resistance and energy absorption capacity improved with increasing loading rate, regardless of whether the foam concrete was confined with CFRP or not;

3. The confining effect induced by the CFRP–foam concrete interaction significantly improved the compression resistance and energy absorption capacity; the compression resistance increased by approximately 10-fold, and the specific energy absorption within a strain of 0.5 increased by approximately 10-fold.

4. The compression resistance and energy absorption capacity of CFRP-confined foam concrete were approximately 300% higher in quasi-static loading and 150% higher in dynamic loading than those of standalone CFRP and foam concrete combined.

## Figures and Tables

**Table 1:** Test results of specimens subjected to quasi-static compression.

Specimen	Mass (g)	Density (kg/m^3^)	Plateau Stress (MPa)	Densification Strain	Specific Energy Absorption (10^6^ J/m^3^)
FC4-S	42	408	0.18	0.70	0.12
FC5-S	51	507	0.22	0.69	0.15
FC4-C-S	42	418	2.00	0.42	0.83
FC5-C-S	51	507	4.37	0.41	1.74
FC4-C-S-2	38	378	7.33	0.41	2.94

Note: FC = foam concrete; 4 = density around 400 kg/m^3^; 5 = density around 500 kg/m^3^; C = with carbon fiber-reinforced polymer (CFRP) confinement; S = static; 2 = double-layer CFRP.

**Table 2:** Test results of specimens subjected to dynamic compression.

Loading Velocity (m/s)	Specimen	Mass (g)	Density (kg/m^3^)	Plateau Stress (MPa)	Specific Energy Absorption (10^6^ J/m^3^)
1	FC4-D1	37	366	0.25	0.13
	FC5-D1	44	439	0.63	0.27
	FC6-D1	63	625	1.09	0.57
	FC4-C-D1	40	399	4.16	2.23
	FC5-C-D1	47	468	5.94	3.06
	FC6-C-D1	58	574	7.27	/
5	FC4-D2	36	362	0.53	0.24
	FC5-D2	47	469	0.86	0.36
	FC6-D2	60	593	1.03	0.46
	FC4-C-D2	42	412	4.88	2.35
	FC5-C-D2	50	496	6.67	3.28
	FC6-C-D2	59	589	6.81	3.69
10	FC6-D3	60	596	3.23	1.42
	FC6-C-D3	58	573	6.49	3.70

Note 1: FC = foam concrete; 4 = density around 400 kg/m^3^; C = with CFRP confinement; D1 = crushing velocity of 1 m/s; D2 = crushing velocity of 5 m/s; D3 = crushing velocity of 10 m/s. Note 2: the strain was taken as 0.5 for all specimens under dynamic compression, due to the loading capacity of the testing machine. Subsequently, the strain of 0.5, which is usually greater than 0.5 for typical low-density foam concrete, was used to calculate the specific energy absorption.
